# Adding tetrahydrofuran to dilute acid pretreatment provides new insights into substrate changes that greatly enhance biomass deconstruction by *Clostridium thermocellum* and fungal enzymes

**DOI:** 10.1186/s13068-017-0937-3

**Published:** 2017-11-30

**Authors:** Vanessa A. Thomas, Bryon S. Donohoe, Mi Li, Yunqiao Pu, Arthur J. Ragauskas, Rajeev Kumar, Thanh Yen Nguyen, Charles M. Cai, Charles E. Wyman

**Affiliations:** 1 Department of Chemical and Environmental Engineering, Bourns College of Engineering, University of California Riverside, Riverside, CA USA; 2 Center for Environmental Research and Technology (CE-CERT), Bourns College of Engineering, University of California Riverside, Riverside, CA USA; 3 National Renewable Energy Laboratory, Golden, CO USA; 4 Joint Institute of Biological Science, Biosciences Division, Oak Ridge National Laboratory (ORNL), Oak Ridge, TN USA; 5 Department of Chemical & Bimolecular Engineering, Center for Renewable Carbon and Department of Forestry, Wildlife, and Fisheries, University of Tennessee Knoxville, Knoxville, TN USA; 6 Department of Bioengineering, Bourns College of Engineering, University of California Riverside, Riverside, CA USA; 7 BioEnergy Science Center (BESC), Oak Ridge National Laboratory, Oak Ridge, TN USA

**Keywords:** Consolidated bioprocessing, Recalcitrance, Yield, Sugar, Fractionation, Tetrahydrofuran

## Abstract

**Background:**

Consolidated bioprocessing (CBP) by anaerobes, such as *Clostridium thermocellum,* which combine enzyme production, hydrolysis, and fermentation are promising alternatives to historical economic challenges of using fungal enzymes for biological conversion of lignocellulosic biomass. However, limited research has integrated CBP with real pretreated biomass, and understanding how pretreatment impacts subsequent deconstruction by CBP vs. fungal enzymes can provide valuable insights into CBP and suggest other novel biomass deconstruction strategies. This study focused on determining the effect of pretreatment by dilute sulfuric acid alone (DA) and with tetrahydrofuran (THF) addition via co-solvent-enhanced lignocellulosic fractionation (CELF) on deconstruction of corn stover and *Populus* with much different recalcitrance by *C. thermocellum* vs. fungal enzymes and changes in pretreated biomass related to these differences.

**Results:**

Coupling CELF fractionation of corn stover and *Populus* with subsequent CBP by the anaerobe *C. thermocellum* completely solubilized polysaccharides left in the pretreated solids within only 48 h without adding enzymes. These results were better than those from the conventional DA followed by either CBP or fungal enzymes or CELF followed by fungal enzyme hydrolysis, especially at viable enzyme loadings. Enzyme adsorption on CELF-pretreated corn stover and CELF-pretreated *Populus* solids were virtually equal, while DA improved the enzyme accessibility for corn stover more than *Populus*. Confocal scanning light microscopy (CSLM), transmission electron microscopy (TEM), and NMR characterization of solids from both pretreatments revealed differences in cell wall structure and lignin composition, location, coalescence, and migration-enhanced digestibility of CELF-pretreated solids.

**Conclusions:**

Adding THF to DA pretreatment (CELF) greatly enhanced deconstruction of corn stover and *Populus* by fungal enzymes and *C. thermocellum* CBP, and the CELF–CBP tandem was agnostic to feedstock recalcitrance. Composition measurements, material balances, cellulase adsorption, and CSLM and TEM imaging revealed adding THF enhanced the enzyme accessibility, cell wall fractures, and cellular dislocation and cell wall delamination. Overall, enhanced deconstruction of CELF solids by enzymes and particularly by *C. thermocellum* could be related to lignin removal and alteration, thereby pointing to these factors being key contributors to biomass recalcitrance as a barrier to low-cost biological conversion to sustainable fuels.

**Electronic supplementary material:**

The online version of this article (doi:10.1186/s13068-017-0937-3) contains supplementary material, which is available to authorized users.

## Background

Combating global climate change requires deployment of energy systems with low net carbon dioxide release [1–3]. Converting the carbon sequestered in lignocellulosic biomass [4–6] such as woody and herbaceous plants and agricultural residues into fuels reduces carbon emissions compared to the current fossil resources as carbon released can be recycled to grow new plants and limited fossil inputs are needed [7–9]. In addition, the US Department of Energy estimated that 0.6–1.6 billion dry tons of non-food biomass could be available annually at an average cost of approximately $60/dry ton [10, 11], enough to displace up to about 80% of the US gasoline use. Furthermore, the unit energy cost of biomass at this price is about that of petroleum at $20/barrel [12–15]. Thus, lignocellulosic biomass stands out as an inexpensive, widely available non-food sustainable resource from which enough liquid fuels could be derived to impact energy demands and reduce atmospheric carbon dioxide accumulation.

Saccharification of lignocellulosic polysaccharide to sugars for fermentation to ethanol and other products remains more expensive than petroleum based liquid fuels due to plant cell wall recalcitrance to chemical, physical, or biological deconstruction [15–17]. *Trichoderma reesei* fungal enzymes have been historically applied to break down plant cell walls [18, 19], but enzymes are estimated to cost ~ $0.70–$1.50/gal ethanol at loadings that achieve viable yields from even relatively low-recalcitrant corn stover [20, 21]. Although tradeoffs among pretreatment types and conditions and fungal enzyme cocktails and loadings have been researched for various lignocellulosic feedstocks [22–26], enzyme costs remain too high [20, 27, 28], and it is desirable to explore new routes to achieving high yields from biological deconstruction.

Consolidated bioprocessing (CBP) by anaerobes such as *Clostridium thermocellum* that produce cellulolytic enzymes and ferment sugars released is a promising alternative to separate fungal enzyme production and subsequent hydrolysis [29–34]. Research on CBP by *C. thermocellum* and other organisms is progressing at various laboratories to achieve industrially relevant ethanol selectivities (> 90%), titers (> 50 g/L), and yields (> 90%). For example, the Lynd’s group at Dartmouth College, NH recently showed that more than 20 g/L of ethanol can be produced from Avicel cellulose with a yield of about 75% of the theoretical maximum [35]. However, little attention has yet to be given to integration of CBP organisms with pretreated real biomass, and it is important to understand whether CBP can realize deconstruction yields competitive with those from conventional pretreatment followed by fungal enzyme hydrolysis and the robustness of CBP to changes in feedstock type [36, 37]. In this study, the following two distinctive pretreatments were applied to two substrates with much different recalcitrance, corn stover and *Populus*, to meet these objectives: (1) conventional dilute acid pretreatment in light of its previously shown versatility with multiple feedstocks [26, 38, 39] and its favored position [40, 41]; and (2) a new pretreatment that applies a miscible solution of tetrahydrofuran (THF) with dilute acid in a technology labeled co-solvent enhanced lignocellulosic fractionation [42]. References to the two are labeled as DA and CELF, respectively, throughout this paper. CELF was chosen to understand how separating a large fraction of major biomass components from one another influences deconstruction of these diverse feedstocks by enzymes and *C. thermocellum* compared to use of dilute acid alone [43]. Although other solvents such as ethanol or methanol (i.e., organosolv that has been studied for over 30 years), newly described gamma valerolactone (GVL), and recent reemergence of ionic liquids could have been employed as pretreatments in this study with likely similar results [25, 44], CELF was selected to take advantage of our extensive experience with optimizing this technology. It is recognized that CELF, as for other solvent pretreatments, has yet to be proven to substantially lower overall process costs or increase revenues through lignin valorization, but THF has important advantages relative to other solvent pretreatments, including low boiling point (66 °C), a high azeotrope concentration in water (95% w/w), which facilitate 97% commercial recovery [45], multiple routes to recovery and recycle, and production from xylose at high yields that can facilitate sustainable replenishment [46]. In addition to determining performance of *C. thermocellum* CBP applied to solids produced by pretreated feedstocks for the first time, enzyme adsorption, changes in biomass and lignin composition, and stereomicroscopy, confocal scanning light microscopy (CSLM), and transmission electron microscopy (TEM) imaging were applied to identify distinctive features of the solids produced by CELF and DA pretreatments of each feedstock that could explain deconstruction differences between CBP and fungal enzyme systems and thereby provide valuable insights that can suggest novel routes to lower the cost of biomass conversion to fuels.

## Results and discussion

### CELF and DA deconstruction of corn stover and poplar wood

**Fig. 1:**
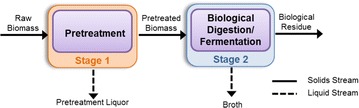
Material flow for dilute acid and CELF pretreatments of corn stover and poplar wood followed by biological deconstruction mediated of the pretreated and washed solids by *Clostridium thermocellum* CBP at 60 °C or fungal enzymes at 50 °C

The BioEnergy Science Center (BESC) through Oak Ridge National Laboratory (ORNL, Oak Ridge, TN) and the National Renewable Energy Laboratory (NREL, Golden, CO) provided BESC standard *Populus* (*Populus trichocarpa*) and corn stover, *Zea mays*, respectively, with compositions of each being reported in the Additional file 1. As illustrated in Fig. 1, CELF and conventional DA [40, 47–49] pretreatments were applied to each feedstock followed by breakdown of the pretreated solids by fungal enzymes over a range of loadings or *C. thermocellum* CBP without enzyme supplementation. Based on our experience in optimizing DA and CELF, both pretreatments were with 0.5 wt% sulfuric acid in water but with addition of equal THF volumes to the acid/water solution for CELF [42]. Lower solids loadings than desired commercially were used to avoid mass-transfer limitations that otherwise can confound the main findings. Combinations of pretreatment temperatures and times were defined to maximize total glucose plus xylose yields from each substrate from the combined pretreatment (Stage 1 in Fig. 1) and subsequent hydrolysis (Stage 2 in Fig. 1) of washed pretreated solids by Accellerase® 1500 enzyme [27, 42]. For the latter, 100 mg-protein/g-glucan was needed to maximize total sugar yields from *Populus*, but 15 mg-protein/g-glucan was adequate for corn stover due to its lower recalcitrance [26]. For DA, 160 °C for 25 min gave the highest combined total sugar yields from *Populus*, while the one for 20 min at 160 °C realized this goal for less-recalcitrant corn stover. For CELF of *Populus*, 160 °C achieved the highest Stage 1 plus Stage 2 total sugar yields but in just 15 min. However, for CELF of corn stover, 150 °C for 25 min achieved the maximum sugar yield [42]. At these conditions, DA removed less than 2% of Klason-lignin in both raw materials, while CELF delignified 82.6 and 75.6% of *Populus* and corn stover, respectively. DA pretreatment hydrolyzed 92.4 and 90.2% of xylan from *Populus* and corn stover, respectively, whereas CELF removed 89.5 and 95.4%, respectively. Over 90% of glucan remained in solids after both pretreatments. Overall, removals of xylan, glucan, and lignin by both DA and CELF were consistent between the two feedstocks, but CELF solids had a considerably higher percent glucan due to greater lignin solubilization. Additional file 1: Table S1 summarizes compositions of solids from each pretreatment at conditions that maximized sugar yields.

### Biological deconstruction of CELF- and DA-pretreated solids

Solids from DA and CELF at the conditions above were washed thoroughly to remove THF and other solubles and hydrolyzed at initial glucan loadings of 5 g/L of glucan. Fungal enzymatic hydrolysis experiments were run in triplicates at 50 °C with enzyme loadings of 2, 5, and 15 mg of Accellerase® 1500 protein/g glucan in biomass before pretreatment. Because these enzyme loadings are projected to cost about $0.10, 0.25, and 0.75/gallon ethanol, respectively, only the lowest is likely to be economically viable [20, 50]. Solids from each pretreatment at optimal conditions were deconstructed in duplicate experiments by *C. thermocellum* at 60 °C for the solids loading of 5 g glucan/L for up to 168 h. Although higher solids levels are desired commercially, low solids loadings were used to focus on deconstruction and avoid *C. thermocellum* inhibition by pretreatment products, culture conditions, and/or hydrolysis products, the latter being also true for fungal systems [24, 51, 52].

**Fig. 2:**
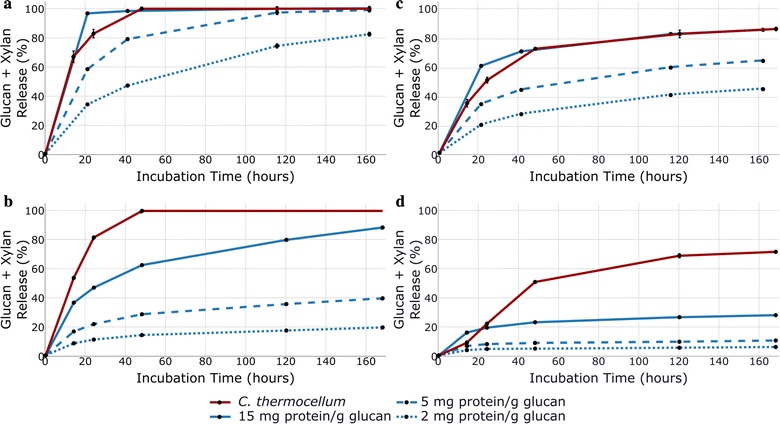
Glucan plus xylan releases from hydrolysis of solids produced by CELF pretreatment of (**a**) corn stover and (**b**) *Populus* and DA pretreatment of (**c**) corn stover and (**d**) *Populus* by fungal enzymes at 50 °C at loadings of 2, 5, and 15 mg of total enzyme protein/g glucan in biomass before deconstruction and by *C. thermocellum* (2% v/v inoculum) at 60 °C. Because *C. thermocellum* fermentation of CELF-pretreated solids was complete in 48 h, its 120- and 168-h time points are extensions of 48-h sugar release. All anaerobic digestion and enzymatic hydrolysis experiments were run in duplicate with mean values shown. Error bars in the graph are one standard deviation. The sugar release for enzymatic hydrolysis refers to sugars recovered in the solution as determined by direct measurement. Sugar release for CBP refers to the amount of sugars solubilized as determined by analysis of the carbohydrates in the residual solids

Figure 2 summarizes how corn stover vs. *Populus* recalcitrance and DA vs. CELF impacted the time course and final yields for deconstruction at three loadings of fungal enzymes vs. *C. thermocellum*. As expected, Fig. 2 demonstrates that corn stover was more amenable to deconstruction than *Populus* for fungal hydrolysis of DA or CELF solids. However, comparing Fig. 2a–d shows that CELF solids were far more easily deconstructed than DA solids regardless of feedstock or biological system. Furthermore, Fig. 2 shows that only the highest fungal enzyme loadings could release as much glucan and xylan from solids produced by DA of corn stover as *C. thermocellum*, while *C. thermocellum* clearly surpassed sugar release by application of even the highest fungal enzyme loadings to solids produced by DA or CELF pretreatments of *Populus*. CELF-pretreated corn stover solids were highly digestible even at 2 mg protein with > 80% glucan plus xylan yield after 7 days; however, consistent with our previous findings, achieving an approximately 95% yield required longer incubation times of 14 days [42]. Most strikingly, CELF followed by *C. thermocellum* virtually completely deconstructed solids from CELF-pretreated corn stover and *Populus* in just 2 days, eliminating differences in polysaccharide recalcitrance between the two feedstocks. Thus, in addition to high yields in shorter times, the CELF–CBP tandem was virtually unaffected by differences in feedstock recalcitrance while DA followed by fungal hydrolysis was.

### Substrate accessibility and enzyme effectiveness

As Additional file 1: Figures S1 and S2 show, CELF followed by *C. thermocellum* CBP achieved near-theoretical glucan and xylan releases from both corn stover and *Populus*, thus overcoming recalcitrance. Because pretreated solids composition and material balances showed similar hemicellulose removal but major differences in lignin solubilization, the much lower lignin content of CELF-pretreated solids (Additional file 1: Table S1) appeared to promote polysaccharide deconstruction.

**Fig. 3:**
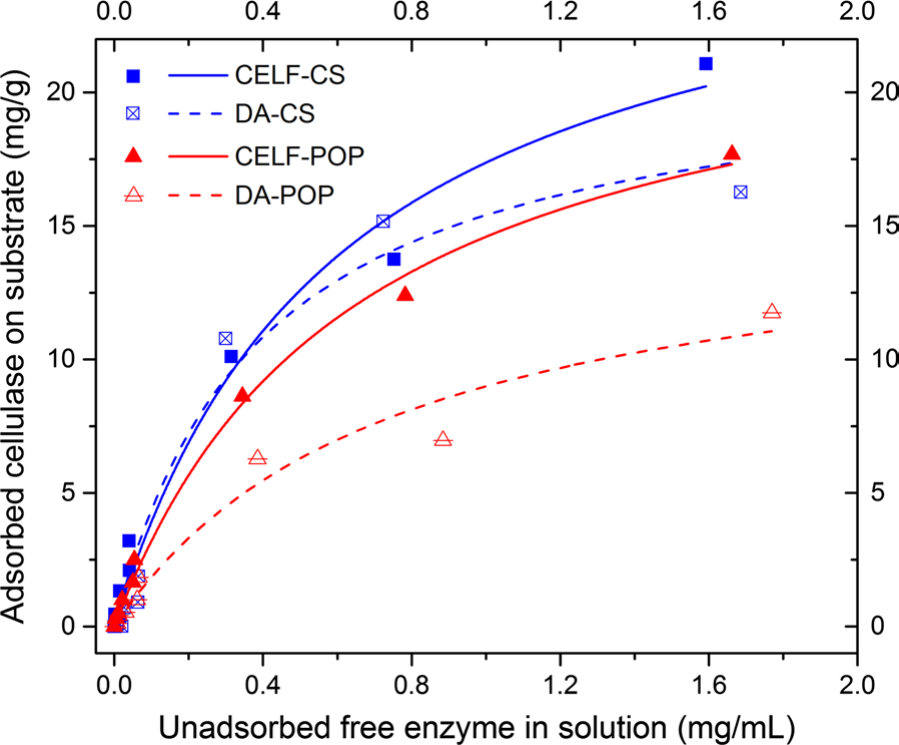
The amount of cellulase adsorbed at 4 °C on solids from CELF and DA pretreatments of corn stover (CELF-CS and DA-CS, respectively) and *Populus* (CELF-POP and DA-POP, respectively) as a function of cellulase remaining in solution for protein concentrations of 0.01–2 mg/mL. Curve fitting was according to the Langmuir adsorption model. *R* ^2^ values: CELF-CS, 0.98; DA-CS, 0.96; CELF-POP, 1.0; and DA-POP, 0.97

As a next step to understand enhanced deconstruction by CELF, cellulase adsorption on CELF- and DA-pretreated corn stover (CELF-CS and DA-CS, respectively) and *Populus* (CELF-POP and DA-POP) was measured for cellulase concentrations of 0.01–2.0 mg protein/mL [53]. Figure 3 shows that CELF-CS solids adsorbed more cellulase than DA-CS solids at higher enzyme concentrations, while enzyme adsorption was similar at low enzyme concentrations. For *Populus*, differences in enzyme adsorption were far more pronounced and grew more so with the increasing enzyme concentration. Adsorption on solids from CELF pretreatment of both corn stover and *Populus* are almost identical but much different on solids from DA pretreatment of both. Enzyme adsorption similarities on CELF solids align with deconstruction patterns for *C. thermocellum* deconstruction of the two and helps explain why CELF–CBP is agnostic to feedstock recalcitrance. On the other hand, disparities between enzyme adsorption on CELF- and DA-corn stover solids are less than those for *Populus*. Reactions of enzyme with substrates are complex and affected by physiochemical properties such as surface features, cellulose ultrastructure, and lignin and hemicellulose [54, 55]. The larger difference in enzyme adsorption kinetics for *Populus* suggests CELF increased enzyme adsorption more for *Populus* than for corn stover compared with DA.

Langmuir nonlinear regression model parameters for the adsorption curve fits in Fig. 3 revealed that the maximum amount of enzyme adsorbed, *Γ*_max_, was similar for CELF-CS and CELF-POP at 28.0 and 24.0 mg/g biomasses, respectively, but dropped to 21.4 mg/g for DA-CS and even more to only 15.7 mg/g for DA-POP. The greater enzyme adsorption on CELF corn stover and *Populus* solids could be due to their significantly lower lignin content resulting in greater cellulose accessibility that outweighed enzyme adsorption on the much greater lignin content of DA solids. The slight drop in enzyme adsorption capacity of DA-CS solids and reduced yields and rates of deconstruction by enzymes and CBP in Fig. 2 supports the possibility that a meaningful portion of enzymes was tied up nonproductively on lignin. The Langmuir-binding affinity constant *K* that is indicative of enzyme affinity for substrate was very similar for CELF-CS, CELF-POP, and DA-POP at 1.6, 1.5, and 1.3 mL/mg, respectively, but rose to 2.6 for DA-CS. However, the fact that rates and yields from DA-CS were lower than from CELF-CS and CELF-POP in Fig. 2 suggests that binding affinity does not significantly impact deconstruction, consistent with higher lignin content in DA solids nonproductively tying up a significant fraction of enzyme. Overall, the Langmuir parameters suggest that enzyme accessibility to substrate, *Γ*_max_, outweighs the impact of binding affinity, *K,* on biomass deconstruction. One hypothesis to explain this result is that the significant enhancement of lignin removal by CELF compared with DA, while achieving similar hemicellulose removal to DA [55] increased enzyme accessibility to solids and their effectiveness so much that enzyme binding did not limit faster, more complete deconstruction of the CELF solids by enzymes or *C. thermocellum*.

### Specific lignin relocation and removal

**Fig. 4:**
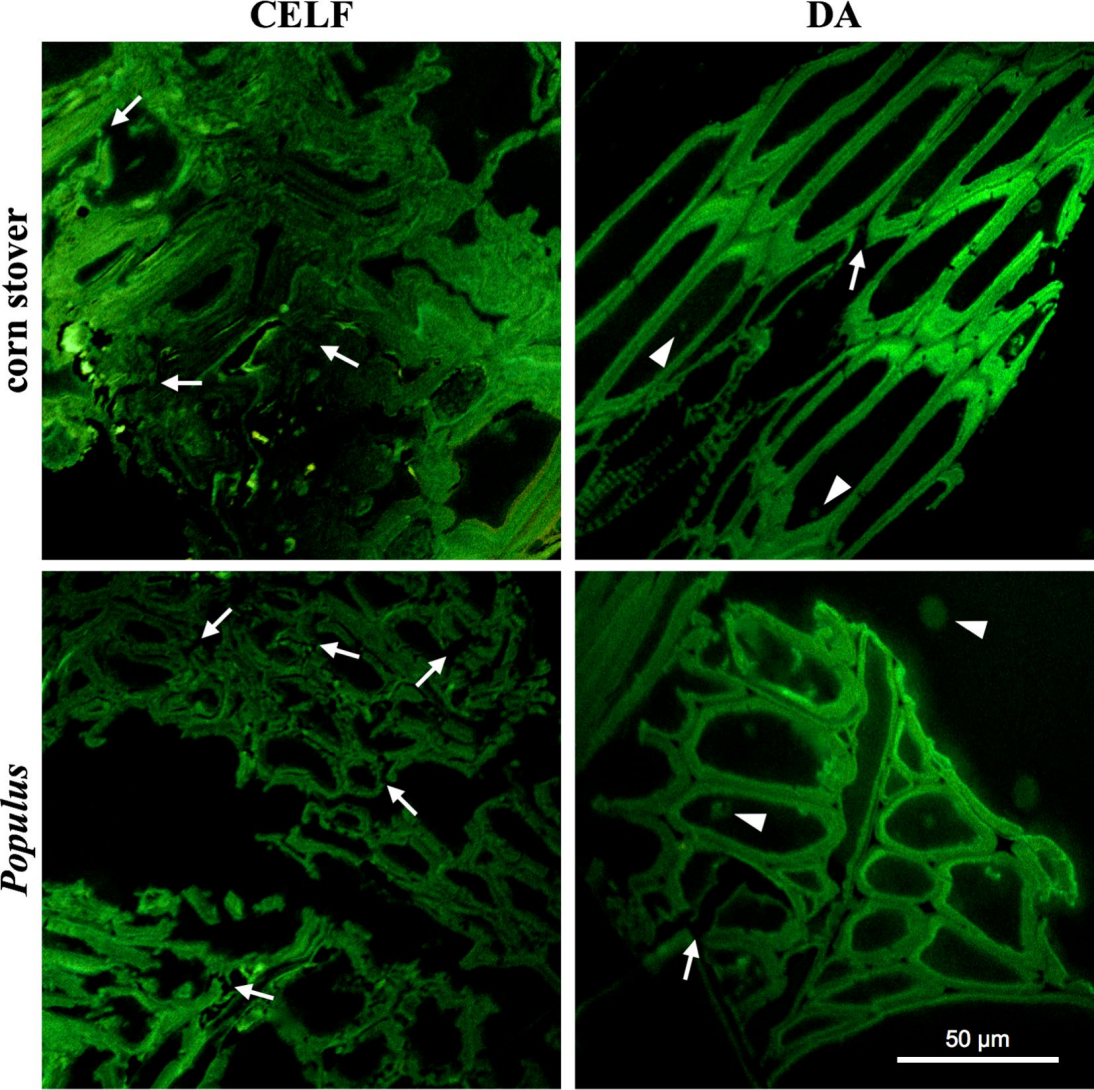
CSLM micrographs of solids produced by CELF (left) and DA (right) pretreatment of corn stover (top) and *Populus* (bottom). Micrographs are of oblique tissue cross sections. Arrows indicate regions of dislocation and fracturing and arrowheads point out coalesced lignin

Confocal scanning light microscopy (CSLM) and transmission electron microscopy (TEM) provided insights into differences in disruption of structural features of solids by CELF vs. DA that could account for enhanced deconstruction by enzymes and *C. thermocellum* and explain why CELF-CBP was agnostic to feedstock recalcitrance. Compared to CSLM images in Figures S3 and S4 for raw corn stover and *Populus*, CSLM micrographs in Fig. 4 show minor dislocation and fracturing (white arrows) for DA-CS and DA-POP. In addition, DA-POP scattered spherical droplets from lignin coalescence (white arrowheads) throughout these images. By comparison, the CSLM micrographs of CELF-CS and CELF-POP in Fig. 4 show much more cell wall delamination, dislocation, and fracturing (white arrows) no droplets as a result of extensive lignin removal. This difference in lignin removal could explain why CELF solids have greater Langmuir enzyme adsorption capacities than DA solids. Although these results do not explain why the binding affinity was so much greater for DA than CELF solids from corn stover or *Populus* or DA-POP, the much greater lignin content of DA compared with CELF solids could bind far more enzyme [56].

**Fig. 5:**
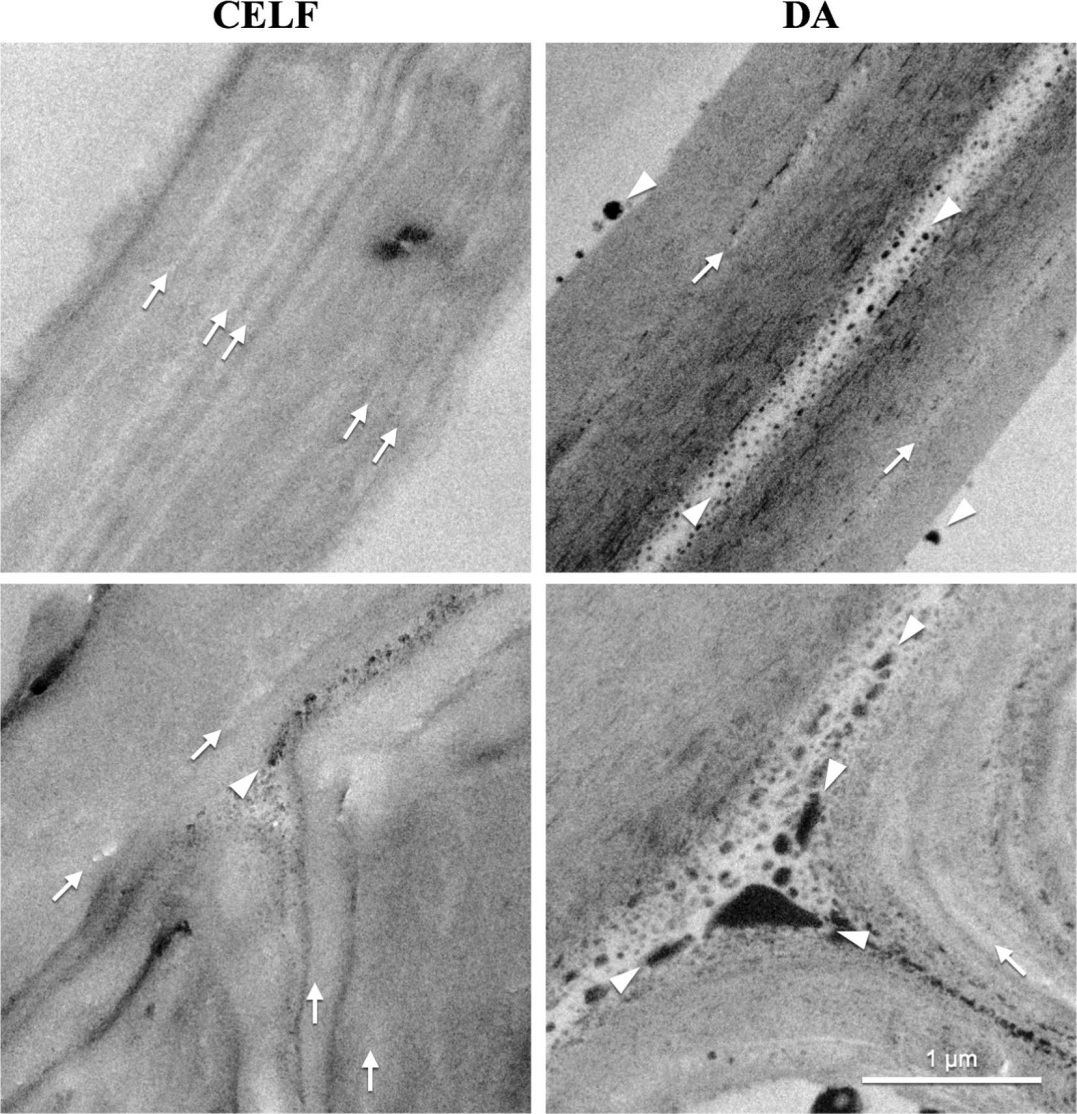
TEM of solids produced by CELF (left) and DA (right) of corn stover. Micrographs of fiber tissue show two adjacent cell walls (top) and intersection of three cell walls (cell wall corners) (bottom) at 1 μm scale. KMnO_4_ staining emphasizes lignin as dark regions. Arrows indicate regions of dislocation and fracturing and arrowheads point out coalesced lignin

**Fig. 6:**
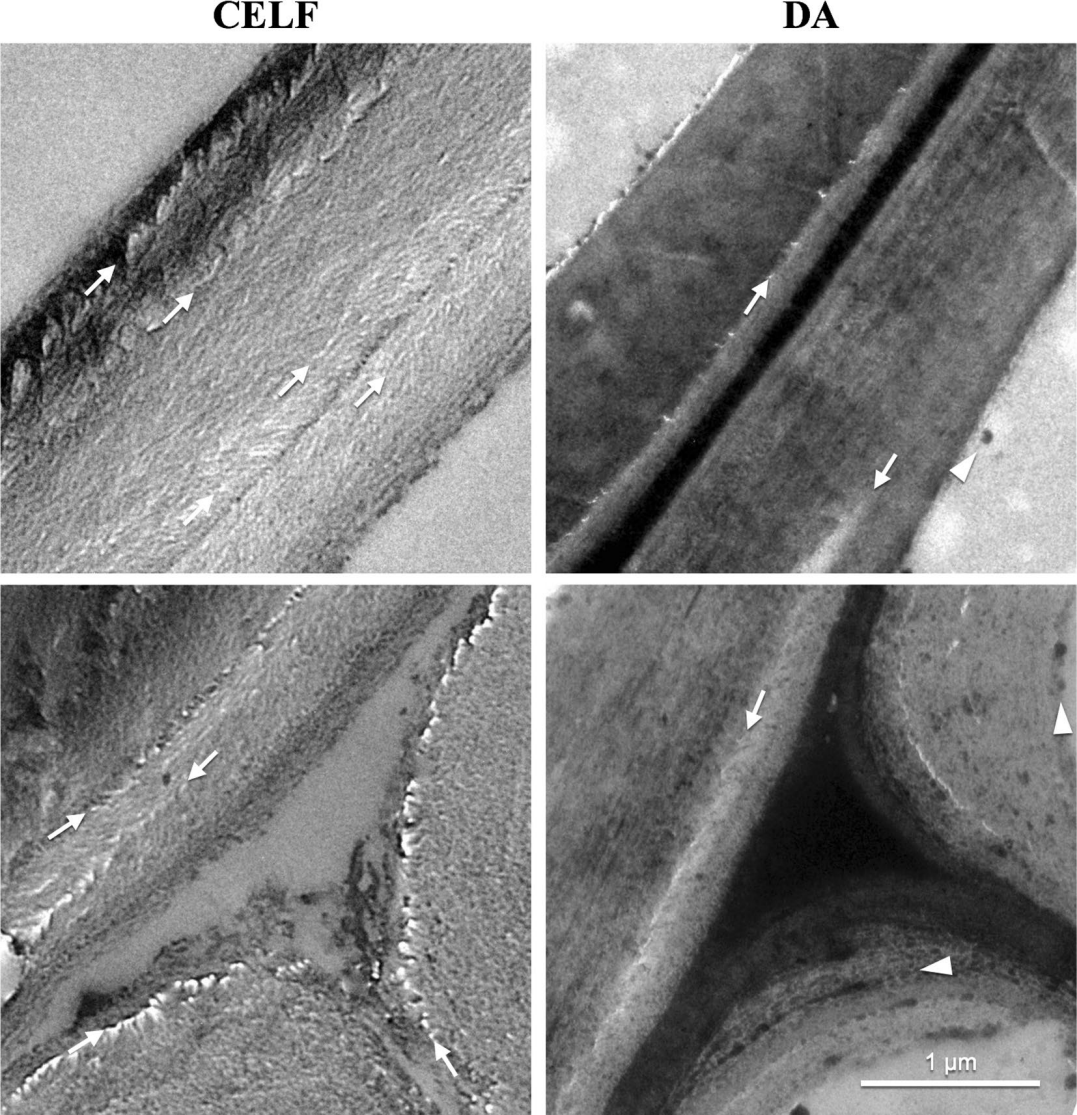
TEM of solids produced by CELF (left) and DA (right) of *Populus* as explained in Fig. 5

TEM micrographs of corn stover fiber and *Populus* cell walls and corners in Figs. 5 and 6 reveal lignin coalescence in the middle lamella and cell wall corners due to migration from the secondary cell wall during DA pretreatment. Lignin coalescence was clearly evident through preferential KMnO_4_ staining of lignin in embedded, sectioned samples (white arrowheads). This interpretation of lignin droplet formation is consistent with previous scanning electron microscopy (SEM) and TEM coupled with electron dispersive spectroscopy (EDS) and NMR analysis that identified electron dense globules as lignin that migrated and coalesced during DA pretreatment of corn stover rind [57]. Additional lignin coalescence was visible in the cell lumen likely due to extrusion from the secondary cell wall through delamination (white arrowheads).

Figures 5 and 6 also show TEM micrographs of cell walls and corners in CELF-CS and CELF-POP solids. The low stain density is consistent with CELF removing significant amounts of lignin from both feedstocks, and TEM micrographs reveal a loose morphology for both. The TEM images also show that CELF increased delamination (white arrows) of corn stover and produced clearly visible cellulose microfibrils and surface erosion of *Populus*. The soft edges of primary and secondary cell walls indicate delamination and loose, splayed fibrils made visible by CELF pretreatment of both feedstocks. Thus, in addition to reinforcing CSLM and enzyme adsorption insights that CELF lignin removal produced, a cleaner surface with greater cellulose accessibility, devoid of lignin deposits that can interfere with enzymes, and had less lignin to nonproductively adsorb enzymes [58], TEM images show that CELF opens up internal cell wall architecture so fungal enzymes and *C. thermocellum* can more readily attack cellulose and achieve greater rates and cellulose deconstruction than from DA. The stereoscope imaging in Figure S5 shows CELF produced smaller fibers from corn stover and more particle swelling in *Populus* compared to DA.

Because lignin composition has been thought to strongly influence biomass recalcitrance [59], HSQC NMR was applied to measure the relative abundance of major syringyl (S) and guaiacyl (G) monolignol subunits and their ratios in both *Populus* and corn stover. In addition, *p*-coumarate (*p*CA), ferulate (FA), and tricin in corn stover; and *p*-hydroxybenzoate (PB) in *Populus* were measured for solids before and after application of the two pretreatments. Key results are summarized here, with more complete data in the Additional file 1. Both CELF and DA pretreatments increased the S lignin fraction and reduced G lignin for both feedstocks. CELF almost completely removed corn stover G units (Additional file 1: Figure S6 and Table S2), while DA only increased the corn stover S/G ratio slightly (from 0.83 to 0.93). For *Populus* in Additional file 1: Figure S7 and Table S2, both CELF and DA increased the S/G ratio slightly from 1.82 in untreated *Populus* to 2.04 and 2.09, respectively. Two-dimensional (2D) NMR lignin spectra revealed that tricin and FA were only detectable at noise level in DA-CS and completely removed for CELF-CS (Additional file 1: Figure S6 and Table S2). Because FA is part of the lignin–carbohydrate complex (LCC) in herbaceous biomass, its removal could lead to LCC breakages that contribute to reduced recalcitrance [60]. In addition, comparison of 2D NMR spectra contours (Additional file 1: Figures S6 and S7) shows CELF removed more *p*CA from corn stover and PB from *Populus* than DA. Preferable removal of lignin moieties by CELF provides new insight into key lignin components that may be responsible for efficient lignin release during biomass pretreatment. In turn, these interpretations are consistent with TEM results in Figs. 4 and 5 that show a cleaner surface in CELF-pretreated substrates due to enhanced lignin removal.

## Conclusions

CELF proved more efficacious than DA for deconstructions of both corn stover and *Populus* in combination with fungal enzymes or *C. thermocellum*. However, the CELF-*C. thermocellum* tandem proved particularly effective in virtually eliminating differences in the native recalcitrances of corn stover and *Populus* with *C. thermocellum* almost completely deconstructing CELF-pretreated solids at nearly identical enhanced rates. CELF–CBP also performed better than CELF followed by hydrolysis with reasonable loadings of fungal enzymes. Composition measurements and material balances, cellulase adsorption, and CSLM and TEM imaging revealed CELF pretreatment rendered corn stover more readily deconstructed by enzymes through high lignin removal, enhanced enzyme accessibility, increased fractures of cell walls, and cellular dislocation and cell wall delamination. In particular, solids produced by CELF pretreatment of corn stover and *Populus* adsorbed more cellulase, *Γ*_max_, than DA solids, despite reducing enzyme affinity, *K*. Interestingly, no clear trend was found between changes in lignin S/G ratio and reduced recalcitrance in that CELF increased the S/G ratio more than DA for corn stover but made a comparable change to DA of *Populus*. Overall, the possible correlation of enhanced deconstruction of CELF solids by fungal enzymes and, particularly *C. thermocellum* with lignin removal and alteration, points to these factors playing key roles in overcoming biomass recalcitrance as a barrier to low-cost biological conversion to sustainable fuels.

## Experimental

### Materials and methods

The BioEnergy Science Center (BESC) through the National Renewable Energy Laboratory (NREL, Golden, CO) and Oak Ridge National Laboratory (ORNL, Oak Ridge, TN) provided corn stover, *Zea mays*, and chipped BESC standard *Populus trichocarpa*, respectively. The corn stover and BESC standard *Populus*, both with moisture contents below 10 w/w%, were knife milled (Thomas-Wiley Laboratory Mill, Model 4, Thomas Scientific, Swedesboro, NJ) through a 1 mm size screen to a particle size < 1 mm. Material that passed through the screen was mixed, divided into 1 gallon bags, and stored at − 20 °C. The small particle size was employed to be consistent with our previous work and avoid confusion by possible mass transfer artifacts due to intraparticle diffusion limitations. Microcrystalline cellulose powder, Avicel® PH-101 from Sigma-Aldrich (St. Louis, MO), was stored at room temperature.

Corn stover and *Populus* were pretreated by dilute acid (DA) and Co-solvent Enhanced Lignocellulosic Fractionation (CELF). Figure 1 illustrates steps in this study, with pretreatment as Stage 1 and fungal enzymatic digestion or CBP fermentation as Stage 2. Pretreated solids were washed before biological digestion at low solids loadings to minimize end-product inhibition of enzymes. Raw and pretreated biomass solids composition, Stage 1 sugar balances, Stage 1 sugar recovery, and Stage 2 sugar release for fungal enzymes and *C. thermocellum* described in the Additional file 1 were used to determine sugar recovery for each feedstock, pretreatment, and biological catalyst combination. The sugar release for enzymatic hydrolysis refers to sugars recovered in the solution, as determined by direct measurement, while sugar release for CBP is the amount of sugars solubilized as determined by analysis of the carbohydrates in the residual solids.

Pretreatments were performed in a 1 L Hastelloy reactor (Parr Instrument Company, Moline, IL) equipped with a pressure gauge, thermocouple (Type K, Omega Engineering, Inc., Stamford, Connecticut), impeller, and electric motor [Pacific Scientific Automation Technology Group (Kollmorgen), Radford, VA]. The reactor was heated to temperature by lowering it into a fluidized sand bath (Model SBL-2D, Techne, Princeton, NJ) maintained at 350–375 °C. The contents were mixed at 180 rpm. Reactor heat up time was that for the temperature to rise from ambient to within 2 °C of the target, the thermocouple accuracy limit. Temperature was controlled by raising and lowering the reactor at the surface of the sand bath. Reaction was stopped by transferring the reactor to a room temperature water bath with cool-down time being from target temperature to 80 °C. The pretreated solids and liquor were separated by vacuum filtration, with liquor stored at − 20 °C. Filtered solids were collected, weighed, and stored at − 20 °C to prevent microbial degradation and compositional changes. Moisture content of solids was measured by oven drying.

Dilute sulfuric acid (DA), and co-solvent enhanced lignocellulosic fractionation (CELF) pretreatments were with 5–10 w/w% solids loading for a 750–800 g total mass. For DA and CELF, untreated biomass was soaked in 0.5 w/w% dilute sulfuric acid and a 50:50 (v:v) mixture of THF:dilute sulfuric acid (0.5 w/w%), respectively, for at least 4 h to allow catalyst penetration.

### Enzymatic hydrolysis

Enzymatic hydrolysis followed the National Renewable Energy Laboratory (NREL, Golden, CO) procedure “Enzymatic Saccharification of Lignocellulosic Biomass” [61]. Loadings of fungal cellulase cocktail Accellerase® 1500 (DuPont Industrial Biosciences, Wilmington, DE; protein concentration ~ 86 mg/mL) were in mg protein/g glucan in raw or pretreated biomass. Protein concentration was determined by a Pierce™ BCA protein assay kit (ThermoFisher Scientific, Pittsburgh, PA). As shown elsewhere, Accellerase® 1500 contains some hemicellulases and auxiliary enzyme activities in addition to cellulase as a major component, [62, 63]. Hydrolysis experiments were run in duplicate for up to 7 days in 125 mL flasks with a working volume of 50 mL at 50 °C and 150 rpm in Multitron shakers (Model AJ125; Infors-HT, Laurel, MD, USA). 50 mM sodium citrate buffer maintained pH at 5.0 ± 0.1. 0.2 g/L sodium azide was added to prevent microbial growth. Enzyme blanks without substrate were incubated with samples to determine any sugar in the enzyme.

### Anaerobic digestion/consolidated bioprocessing

*Clostridium thermocellum* DSM 1313 was from Professor Lee R. Lynd, Dartmouth College (Hanover, NH). Seed inoculum was from a single batch of a mono-colony isolate of exponential phase *C. thermocellum* cultured in MTC medium [64] and Avicel® PH-101 at 60 °C and 180 rpm. Media chemicals were from Sigma-Aldrich (St. Louis, MO) or Fisher Scientific (Pittsburgh, PA). Seed inoculum was divided into 4 mL aliquots and stored at − 80 °C. Freezer stocks were cultured on 5 g Avicel® PH-101 glucan/L using MTC medium (less trace elements and yeast extract) for 2 v/v% inoculum. 50 mL working volumes were loaded with 5 g glucan/L of pretreated biomass and transferred freezer stock cultures. Over a 4 year period, Avicel® controls were run at identical conditions to be sure the inoculum continued to reach ~ 90% glucan release in 24 h. Glucan release was calculated as glucan weight in solution after 24 h relative to the glucan weight loaded initially. To calculate solids dry weight after 24 h, the entire fermentation contents were collected, washed (via vortexing as described later), and oven dried at 105 °C overnight. Details on yield and material balance calculations are presented in the Additional file 1.

Cultures and media were in serum bottles plugged with butyl rubber stoppers (Chemglass Life Sciences, Vineland, NJ) and sealed by aluminum crimps. To make anaerobic, the headspace was flushed with nitrogen gas and then evacuated by a compressor (model ABF63 4B 7RQ, ATB, Vienna, Austria) for 45 s. The flush/evacuation cycle was repeated 15 times. Biomass and substrates were autoclaved at 121 °C for 30 min, and media autoclaved or filter sterilized (0.22 μm filter, Millipore, Billerica, MA) for heat sensitive compounds. Bottle fermentations were maintained at pH 7.0 with MOPS buffer. All the anaerobic digestion experiments were run in duplicate, with mean values reported. Samples were at 12 or 24 h intervals for 7 days.

The entire reactor contents were centrifuged at 2800 rpm to remove liquid for HPLC analysis, and residual solids were washed three times, each with 50 mL of DI water after vortexing solids and water between washings. Residual solids were dried and weighed to determine total mass loss followed by polysaccharide and lignin quantification.

### Structural sugars and lignin quantification

Raw, pretreated, and post CBP solids were analyzed for structural sugars and lignin via NREL procedure “Determination of Structural Carbohydrates and Lignin in Biomass” [65]. Wheat straw (RM 8494) or Eastern Cottonwood (RM 8492) from the National Institute of Standards and Technology (Gaithersburg, MD) were also analyzed as standards. If < 300 mg of solids remained after fermentation, the procedure was scaled down for the available sample weight.

Liquid samples from pretreatment, enzymatic hydrolysis, and fermentation were analyzed for soluble sugar monomers and oligomers by HPLC. To analyze monomers, 30 μL of 10 w/w% sulfuric acid was added to 1 mL enzymatic hydrolysis and fermentation samples to stop reactions, vortexed, and centrifuged to remove solids and cell debris prior to analysis. To quantify oligomers, liquid samples were post hydrolyzed per the NREL procedure “Determination of Structural Carbohydrates and Lignin in Biomass” [65].

A Waters HPLC separations module e2694 with refractive index detector 2414, (Milford, MA) and Aminex HPX-87H column (Bio-Rad, Hercules, CA) eluted with 50 mM sulfuric acid separated cellobiose, glucose, xylose, arabinose, formate, lactate, acetate, levulinic acid, ethanol, 5-HMF, and furfural. Two to five replicates were run for each analysis.

### Cellulase adsorption

Cellulase C2730 (*T. reesei* ATCC 26921, protein content 40 mg/mL, Sigma-Aldrich) adsorption on 2% (w/v)-pretreated solids was at 4 °C in 50 mM citrate buffer (pH 4.8) [66] over concentrations from 0.01 to 2.0 mg protein/mL (0.5–100 mg protein/g solids). The mixture was equilibrated at 4 °C for 2.5 h in 15 min shaking intervals. The supernatant cellulase protein content was determined by the Bradford assay using bovine serum albumin (BSA) as a standard [67]. Cellulase adsorption was calculated as the difference between initial cellulase added and cellulase left in supernatant. Cellulase adsorption on CELF- and DA-pretreated biomass substrates was modeled by the classical Langmuir adsorption isotherm, with the adsorbed enzyme concentration (*Γ*) calculated as:

1 $$\varGamma = \frac{{\varGamma_{\rm{max} } KC}}{1 + KC},$$

where *Γ* is bound enzyme (mg/g substrate), *Γ*_max_ the surface concentration of protein at full coverage (mg/g substrate), *K* the binding affinity constant (mL/mg), and *C* the bulk solution protein concentration (mg/mL) [53].

### Imaging by stereoscopy, CSLM, and TEM

Pretreated biomass and solid residues after *C. thermocellum* CBP were imaged by stereoscopy, CSLM, and TEM. For stereoscopy, a Nikon SMZ1500 stereomicroscope with a Nikon DS-Fi1 CCD camera operated by a Nikon Digital Sight system (Nikon Instruments, Melville, NY) examined biomass particles without further processing [68].

Prior to CSLM or TEM, biomass samples were fixed for 2 × 6 min (with variable power) in 2.5% gluteraldehyde buffered in 0.1 M sodium cacodylate buffer (EMS, Hatfield, PA) under vacuum and dehydrated with increasing acetone concentrations (15, 30, 60, 90, and 3 × 100% acetone) for 1 min at each dilution. Samples were then infiltrated with LR White resin (EMS, Hatfield, PA) by incubating at room temperature (RT) for several hours to overnight in increasing concentrations of resin (30, 60, 90, 3 × 100% resin, diluted in ethanol). Samples were transferred to capsules, and the resin was polymerized at 60 °C overnight. LR White embedded samples were sectioned to ~ 60 nm with a Diatome diamond knife on a Leica EM UTC ultramicrotome (Leica, Wetzlar, Germany). Sections were collected on 0.5% Formvar coated slot grids (SPI Supplies, West Chester, PA).

For CSLM, 300 nm sectioned samples were positioned on glass microscope slides and stained with 0.1% acriflavine, a fluorochrome for lignin detection. Images were captured using a 40X 1.3NA Plan Fluor lens on a Nikon C1 Plus microscope (Nikon, Tokyo, Japan), equipped with a Nikon C1 confocal system operated via Nikon’s EZ-C1 software and using 488 nm laser excitation. For TEM, 60 nm sections placed on grids were post-stained for 6 min with 2% aqueous uranyl acetate and 10 min with 1% KMnO4 to selectively stain lignin. Images were captured by a 4-mega-pixel Gatan UltraScan 1000 camera (Gatan, Pleasanton, CA) on FEI Tecnai G2 20 Twin 200 kV LaB6 TEM (FEI, Hilsboro, OR). Additional details on preparation, microscopy execution, and image capture, curating, processing, and analysis for CSLM and TEM are described elsewhere [69].

### Heteronuclear single quantum coherence (HSQC) 2D-NMR of lignin relative monolignol subunit abundance determination

Samples were freeze dried and extracted by ethanol:toluene (1:2, v/v) via a Soxhlet apparatus before analysis. Lignin samples were isolated by dioxane:water (96:4, v/v) extraction after ball-milling by a Retsch PM 100 planetary mill and treatment with mixed cellulolytic enzymes (Cellic ® CTec2 and HTec2, gifts from Novozyme). Lignin samples obtained were dissolved in DMSO-_d6_ using a Shigemi micro-tube, and lignin spectra were acquired with a 400-MHz Bruker Avance-III spectrometer. HSQC experiments applied a 10-ppm spectra width in F2 (^1^H) dimension with 2048 data points, 210-ppm spectra width in F1 (^13^C) dimension with 256 data points, 1.5-s pulse delay, and a ^1^ *J*_C–H_ coupling constant of 145 Hz. 128 or 320 scans were employed depending on sample concentration. The central DMSO solvent peak (*δ*_C_ 39.5 ppm; *δ*_H_ 2.49 ppm) was used for chemical shift calibration. NMR data were processed using TopSpin 2.1 (Bruker BioSpin) software packages [70].

## Additional file

**Additional file 1.** Additional information.

## Abbreviations

CBP: consolidated bioprocessing by a single organism, in this case, *Clostridium thermocellum* that produces cellulolytic enzymes and ferments the sugars released in a single vessel
CELF: pretreatment with a miscible mixture of tetrahydrofuran (THF) and an equal volume of 0.5% sulfuric acid in water
CELF-CS: solids produced by CELF pretreatment of corn stover
CELF-POP: solids produced by CELF pretreatment of *Populus*
CSLM: confocal scanning light microscopy
DA: pretreatment with dilute sulfuric acid, in this case, 0.5% sulfuric acid at 160 °C
DA-CS: solids produced by DA pretreatment of corn stover
DA-POP: solids produced by DA pretreatment of *Populus*
HSQC: heteronuclear single quantum coherence 2D-NMR
NMR: nuclear magnetic resonance
TEM: transmission electron microscopy
