# Supplementary material for: Adding tetrahydrofuran to dilute acid pretreatment provides new insights into substrate changes that greatly enhance biomass deconstruction by Clostridium thermocellum and fungal enzymes
Source: Biotechnol Biofuels. 2017 Nov 30;10:252. doi: 10.1186/s13068-017-0937-3 (PMC5707920; doi:10.1186/s13068-017-0937-3)
Supplement: Supplementary file 1 — Additional file 1. Additional information. [file 13068_2017_937_MOESM1_ESM.docx]

## **Additional information**

## **Adding Tetrahydrofuran to Dilute Acid Pretreatment Provides New Insights into Substrate Changes that Greatly Enhance Biomass Deconstruction by *Clostridium thermocellum* and Fungal Enzymes**

Vanessa Thomas^1,2,7^, Bryon S. Donohoe^3,7^, Mi Li^4, 7^, Yunqiao Pu^4,7^, Arthur J. Ragauskas^4,5,7^, Rajeev Kumar^2,7^, Thanh Yen Nguyen^2,6^, Charles M. Cai^2,7^, Charles E. Wyman^1,2,6,7*^

^1^Department of Chemical and Environmental Engineering, Bourns College of Engineering, University of California Riverside, Riverside, CA 92521, ^2^Center for Environmental Research and Technology (CE-CERT), Bourns College of Engineering, University of California Riverside, Riverside, CA 92507, ^3^National Renewable Energy Laboratory, Golden, CO 80401, ^4^Joint Institute of Biological Science, Biosciences Division, Oak Ridge National Laboratory (ORNL), Oak Ridge, TN 37830, ^5^Department of Chemical & Bimolecular Engineering, Center for Renewable Carbon and Department of Forestry, Wildlife, and Fisheries, University of Tennessee Knoxville, Knoxville, TN 37996, ^6^Department of Bioengineering, Bourns College of Engineering, University of California Riverside, Riverside, CA 92521, ^7^BioEnergy Science Center (BESC), Oak Ridge National Laboratory, Oak Ridge, TN 37830

*****Corresponding author: Charles E. Wyman; Email: [cewyman@engr.ucr.edu](mailto:cewyman@engr.ucr.edu), Phone: (951) 781-5703

## **Materials and Methods**

### **Sugar release calculations**

Sugar release refers to the amount of sugar solubilized from the solid biomass into the liquid by enzymatic digestion or consumed by the organism during fermentation. For the case of either biological catalyst, polysaccharide hydrolysis was considered as:

or more simply,

where A is the total amount of insoluble polysaccharides originally present in the biomass given in terms of sugar monomer equivalent and B is the total amount of soluble sugar monomer that results from hydrolysis. The subscripts *(s)* and *(l)* refer to solid and liquid, respectively. It is assumed that sugar does not degrade during biological deconstruction, and B, therefore, does not react further.

For free fungal enzymatic hydrolysis, sugar release was calculated as the percent sugar yield Y(t), defined as the amount of sugar released by enzymes into solution at time t relative to the amount of sugar originally present prior to enzymatic hydrolysis:

where *n* represents an amount of sugar in grams as either a polysaccharide, *n_A_*, or monomer *n_B_*, and *t* represents time. Sugar release by *C. thermocellum* produces a variety of products, some of which cannot be readily measured, thus, its performance was calculated in terms of polysaccharide percent conversion, X(t), which was defined in terms of the amount of polysaccharide that was removed from the solid between time *t=0* and *t*, relative to the original amount of polysaccharide present in the solids at time *t=0*:

Note that the conversion is defined only in terms of *n_A_*, the amount of insoluble polysaccharide in the solid, while the yield is defined in terms of both *n_A_* and *n_B_*, the amount of sugar monomer found in the liquid. When a low solids loading is used, as is the case with this research, we can assume that end-product inhibition of the enzymes by sugars is negligible. Because we found that fungal enzymes completely hydrolyze dissolved polysaccharides to sugar monomers with no accumulation of oligomers, calculating digestion by yield is appropriate as both *n_A_* and *n_B_*, are measurable. However, since a CBP organism performs both hydrolysis and fermentation, the sugars released into solution are also consumed, therefore, *n_B_* is not quantifiable. Measuring polysaccharide conversion for *C. thermocellum* is the more appropriate route for calculating reaction progress as *n_A_* is the only measurable quantity and the only term required in equation (4).

Yield and conversion are related through selectivity, $S$, as follows:

However, for a linear reaction which forms only a single product, as in equation (2),

and,

Thus, yield and conversion are equivalent in the case of biological deconstruction of biomass with no sugar degradation. The only variance between yield and conversion is how the reaction progress is measured: either by sugar appearance in the liquid or polysaccharide disappearance from the solid, for yield and conversion, respectively, relative to the amount of polysaccharide originally present in the solid. In order to compare saccharification by enzymatic hydrolysis directly to that by fermentation, the single term, sugar recovery,$R$, can be explicitly defined as:

Here *R* is interpreted as either yield or conversion, depending on the biological system applied.

### **Stage 1 sugar material balance calculations**

Glucan and xylan material balances were applied around the pretreatment step, Stage 1, to validate the yields calculated and determine the fraction of the sugars lost to degradation. The generalized total material balance for pretreatment can be expressed as:

where $g$ and $x$ are the masses of glucan and xylan in the raw biomass, respectively, and $g'$, $x'$, and $d'$ are the masses of glucan, xylan, and sugar degradation products present after pretreatment, respectively, in grams. The subscripts *(s)* and *(l)* indicate the solid and liquid phase, respectively. For a glucan or xylan only material balances, only the g or x symbols, respectively, in equation 9 were applied. The sugar degradation products can be calculated as the sum of individual compounds that resulted from glucan and xylan degradation:

where $d_{i}$ is the amount of an individual degradation product in equivalent mass of glucan or xylan, and *n* is the total number of degradation products quantified. In this study, the degradation products quantified were levulinic acid, formic acid, 5-HMF, and furfural. Pretreated solids were analyzed for structural polysaccharides while pretreatment liquors were analyzed for oligosaccharides, monomers, and degradation products to account for the total amount of glucan and xylan in the raw biomass prior to pretreatment.

### **Stage 2 sugar material balance calculations**

Stage 2 material balances were calculated in a similar manner to those for Stage 1:

However, now $g$ and $x$ are the amounts of glucan and xylan in the pretreated biomass, respectively, and $g'$ and $x'$ are the amounts of glucan and xylan present after biological digestion (Stage 2), respectively, in grams. The subscripts *(s)* and *(l)* indicate the solid and liquid phase, respectively.

## **Results and Discussion**

**Composition of solids following DA and CELF pretreatments of corn stover and *Populus***

Table S1 reports the compositions of *Populus* and corn stover solids before and after DA and CELF pretreatments. Based on material balance for *Populus* and corn stover, approximately 100% and 98% of the glucan, respectively, and 92% of the xylan from either feedstock could be accounted for in the solids and liquid streams from CELF pretreatment. Total sugar recovery was somewhat lower for DA pretreatment, for which approximately 99% of the glucan from *Populus* and 95% from corn stover, and 91% of the xylan from either one could be accounted for after pretreatment (Stage 1). Thus, most of the glucose and xylose was preserved in the liquids and solids resulting from these pretreatments of corn stover and *Populus* due to limited degradation, and those left in the pretreated solids were accessible for nearly complete hydrolysis at high enzyme loadings.

| **Table S1.** Compositions of *Populus* and corn stover solids before and after DA and CELF pretreatments. | | | | | |
| --- | --- | --- | --- | --- | --- |
| Biomass type and pretreatment | | Glucan (%) | Xylan (%) | Klason lignin (%) | Other (%) |
| *Populus* | Raw | 46.8 | 15.9 | 21.8 | 15.5 |
|  | DA* | 64.0 | 1.8 | 31.6 | 2.6 |
|  | CELF** | 85.5 | 3.3 | 7.5 | 3.7 |
| Corn stover | Raw | 36.1 | 28.5 | 14.5 | 20.9 |
|  | DA | 54.1 | 4.9 | 25.6 | 15.4 |
|  | CELF | 74.8 | 3.1 | 8.3 | 13.8 |
| *DA- dilute acid; CELF- Co-solvent Enhanced Lignocellulosic Fractionation  Standard deviations for all values were within ± 2%, which was the error associated with repeatability of the procedure for determining structural sugars and lignin | | | | | |

**Release of xylan plus glucan by CELF and DA pretreatments of corn stover and *Populus* and from deconstruction of pretreated solids by fungal enzymes or *Clostridium thermocellum***

Figures S1 and S2 show the total amount of xylan and glucan released from corn stover and *Populus* from the combined operations of CELF or DA pretreatment (Stage 1) followed by biological conversion (Stage 2) at various loadings of fungal enzymes or by CBP with *Clostridium thermocellum* acting alone without added enzymes. These results point out the greater recalcitrance of *Populus* to deconstruction by fungal enzymes compared to corn stover, higher yields from CELF pretreatment compared to DA at the same loadings of fungal enzymes, and superior yields from CELF pretreated solids at lower, more economically attractive enzyme loadings. Of particular importance, the data demonstrate that the CELF/CBP combination is feedstock agnostic in that similar yields are achieved with *Populus* and corn stover despite their widely different recalcitrance to fungal enzyme deconstruction and that CELF/CBP achieves these high yields for only a 2 day CBP fermentation.

**Figure S1.** Stage 1 and Stage 2 glucan plus xylan release from corn stover. Stage 1 refers to either CELF or DA pretreatment and Stage 2 refers to either enzymatic hydrolysis at enzyme loadings of 2, 5, or 15 mg protein of cellulase/g glucan in raw biomass or *C. thermocellum* CBP at 2% (v/v). Stage 2 glucan plus xylan release is reported at the maximum sugar release time of 48 hours for CELF-CBP and 168 hours for the other combinations of CELF and DA pretreatments with fungal enzymes or CBP.

**Figure S2**. Stage 1 and Stage 2 glucan plus xylan release from *Populus*. Stage 1 refers to either CELF or DA pretreatment and Stage 2 refers to either enzymatic hydrolysis at enzyme loadings of 2, 5, or 15 mg protein of cellulase/g glucan in raw biomass or *C. thermocellum* CBP at 2% (v/v). Stage 2 glucan plus xylan release is reported at the maximum sugar release time of 48 hours for CELF-CBP and 168 hours for the other combinations of CELF and DA pretreatments with fungal enzymes or CBP.

**Imaging of solids produced by CELF and DA pretreatment of corn stover and *Populus***

Raw, milled corn stover was imaged by stereoscopy, as shown in Figure S3, to serve as a control set of micrographs. Stereoscope images showed the range of particle size, shape, and color. A 1 mm particle size screen was used to mill the corn stover, as evident by the size and shape of the particles. The CSLM micrographs show largely intact regions of tissue with some degree of cell wall fracture, likely from milling, with uniform cell wall thickness. The TEM micrographs show cell walls that are full and intact. Lignin is evenly distributed in homogeneous, parallel layers seen as thin black lines running through the cell wall with occasional collections of high density lignin appearing as black spots.


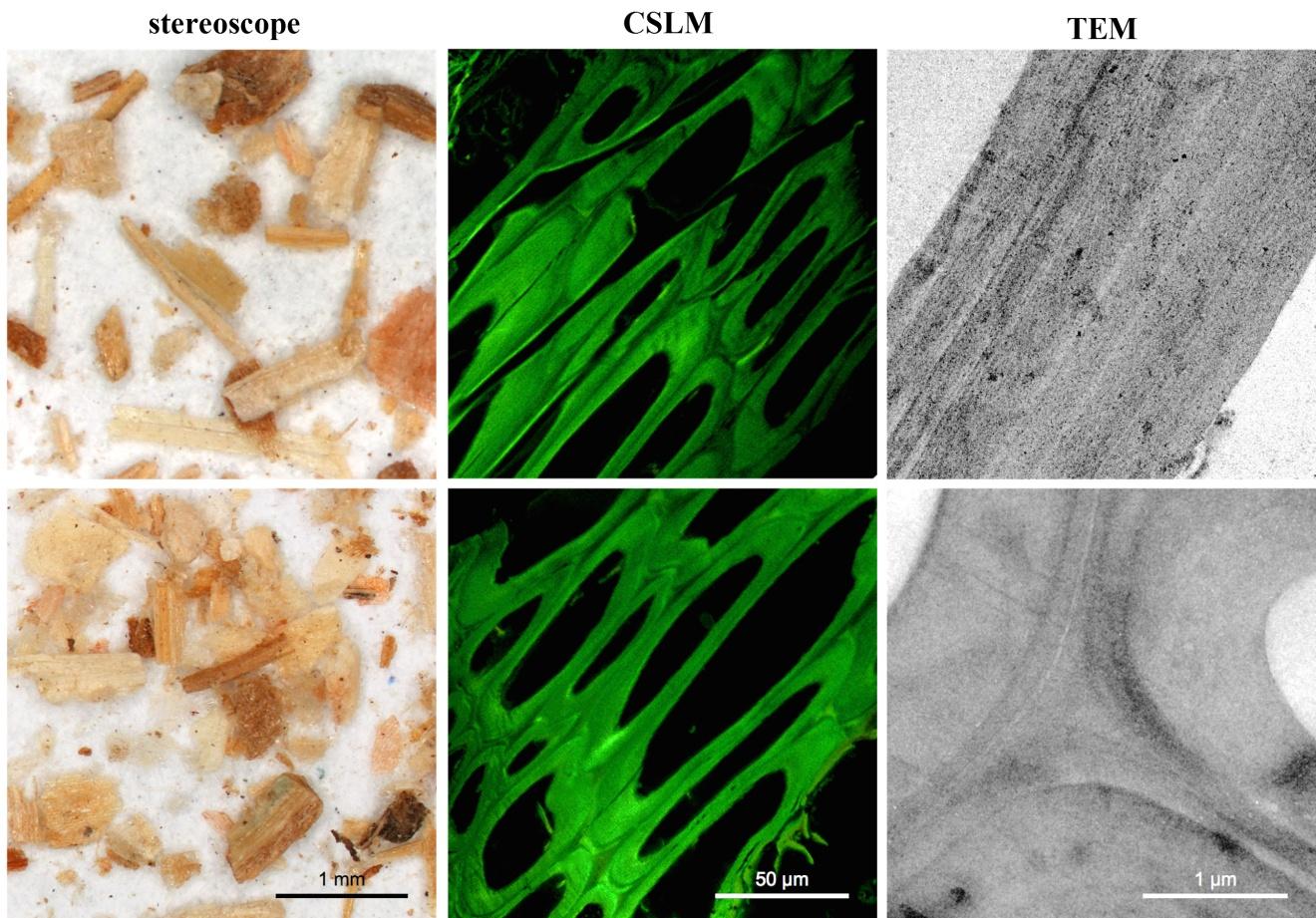


**Figure S3.** Control stereoscopic, CSLM, and TEM micrograph images of raw, milled corn stover showing biomass particles at the tissue, cellular, and organelle scale, respectively. CSLM micrographs show oblique cross sections of tissue at a scale of 50 μm while TEM shows two adjacent cell walls (top) and the intersection of three cell walls, known as a cell corner, (bottom) at a scale of 1 μm. Tissue was stained with KMnO_4_ for TEM images to show the location of lignin as dark regions.

As shown in Figure S4, raw, milled *Populus* was also imaged by stereoscopy, CSLM, and TEM to serve as a control set of micrographs. The stereoscope images showed the range of particle size, shape, and color. The size and shape of the *Populus* particles are as would be expected for milling through a 1 mm size screen. The CSLM micrographs show largely intact regions of tissue with some degree of fractionation, likely from milling, with uniform cell wall thickness. The TEM micrographs show full and intact cell walls. Lignin is evenly distributed in homogeneous, parallel layers seen as thin black lines running through the cell wall with occasional collections of high density lignin appearing as black spots.


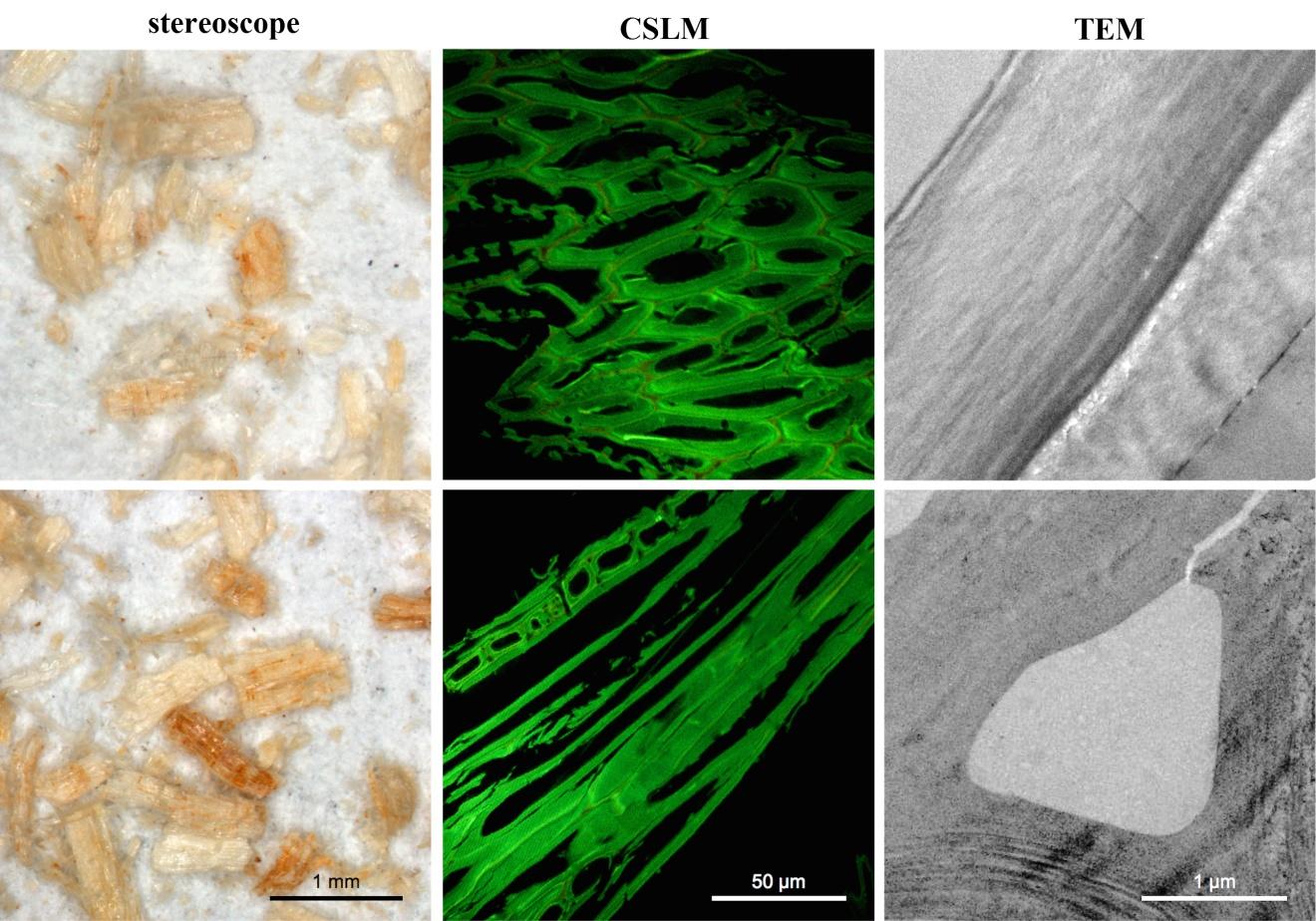


**Figure S4.** Control stereoscopic, CSLM, and TEM micrograph images of raw, milled *Populus* showing biomass particles at the tissue, cellular, and organelle scale, respectively. CSLM micrographs show oblique cross sections of tissue at a scale of 50 μm while TEM shows two adjacent cell walls (top) and the intersection of three cell walls, known as a cell corner, (bottom) at a scale of 1 μm. Tissue was stained with KMnO_4_ for TEM images to emphasize the location of lignin as dark regions.

Comparing the stereoscope images of DA pretreated corn stover in Figure S5 to those for unpretreated corn stover in Figure S3 reveals that DA frayed and opened up the solids and generated more fine material. Stereoscope images of CELF pretreated corn stover solids in Figure S5 show that CELF produced smaller fibers than DA.


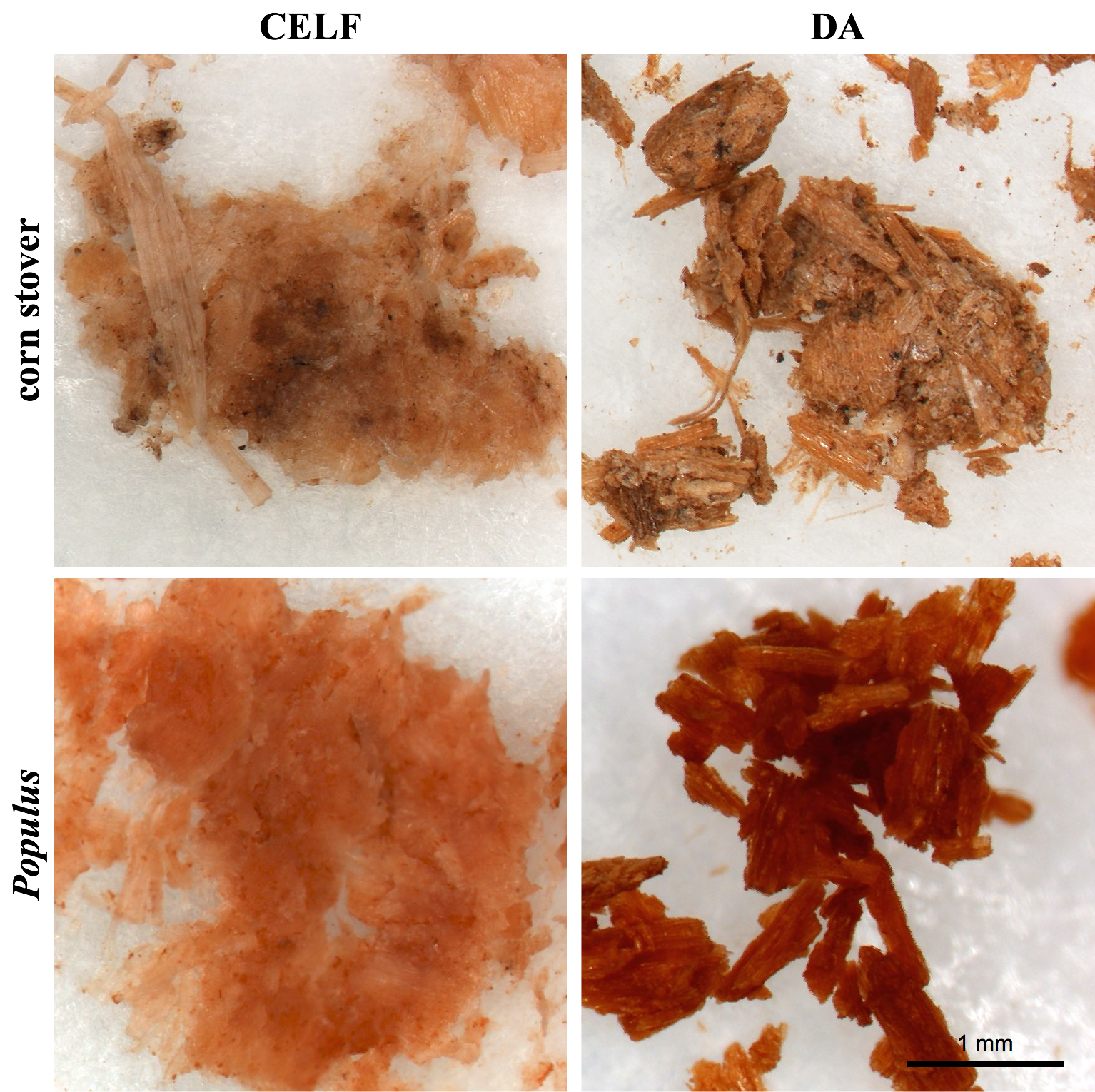


**Figure S5.** Stereoscope images of solids produced by CELF (left) and DA (right) pretreatments of corn stover (top) and *Populus* (bottom).

Figure S5 also shows stereoscopy images of solids produced by dilute sulfuric acid and CELF pretreatments of *Populus*. These micrographs provide evidence of particle swelling caused by CELF and color change and clumping caused by the DA pretreatment.

**Characterization of lignin left in solids following CELF and dilute acid pretreatment of corn stover and *Populus***

Table S2 provides measured values of the relative abundance of syringyl (S) and guaiacyl (G) subunits for extractive-free corn stover and *Populus* along with the corresponding S/G ratio. The table also includes *p*-coumarate (*p*CA), ferulate (FA), and tricin values for corn stover, and *p*-hydroxybenzoate (PB) measurements for *Populus*. Figures S6 and S7 provide the corresponding 2D NMR spectra of corn stover and *Populus*, respectively, lignin aromatic regions that reveal its subunits.

| **Table S2.** Relative abundance of syringyl (S), guaiacyl (G), *p*-coumarate (*p*CA), ferulate (FA), tricin, and *p*-hydroxybenzoate (PB) lignin subunits and ratio of S to G (S/G) in extractive-free corn stover and *Populus* samples. | | | | | | | | |
| --- | --- | --- | --- | --- | --- | --- | --- | --- |
| Corn Stover | S(%) | S’(%) | G(%) | G’ (%) | pCA (%)**^b^** | FA(%)**^b^** | Tricin (%) | S/G |
| Raw | 45.0 | ND | 54.5 | ND | 84.1 | 14.9 | 5.7 | 0.83 |
| CELF pretreated | ~100 | ND | ND | ND | 147.7 | ND | ND | NA^c^ |
| DA pretreated | 45.5 | 2.6 | 42.5 | 9.5 | 91.2 | ND | ND | 0.93 |
|  | | | | | | | | |
| *Populus* | S(%) | S’(%) | G(%) | G’(%) | PB(%)^a^ | | | S/G |
| Raw | 60.1 | 4.3 | 34.2 | 1.3 | 15.0 | | | 1.82 |
| CELF pretreated | 67.1 | ND | 32.9 | ND | 2.8 | | | 2.04 |
| DA pretreated | 64.4 | 3.3 | 29.6 | 2.8 | 18.1 | | | 2.09 |

Note: ^a^ PB(%) is the percentage of the *p*-hydroxybenzoate on a lignin (S+G) basis. ^b^ *p*CA(%) and FA(%) are percentages of the *p*-coumarate and ferulate on the basis of lignin S+G, respectively. S’: oxidized syringyl subunits. G’: oxidized guaiacyl subunits. ^c^ NA: not applicable (S/G value becomes not available as the G signal is at the noise level). ND: not detectable.


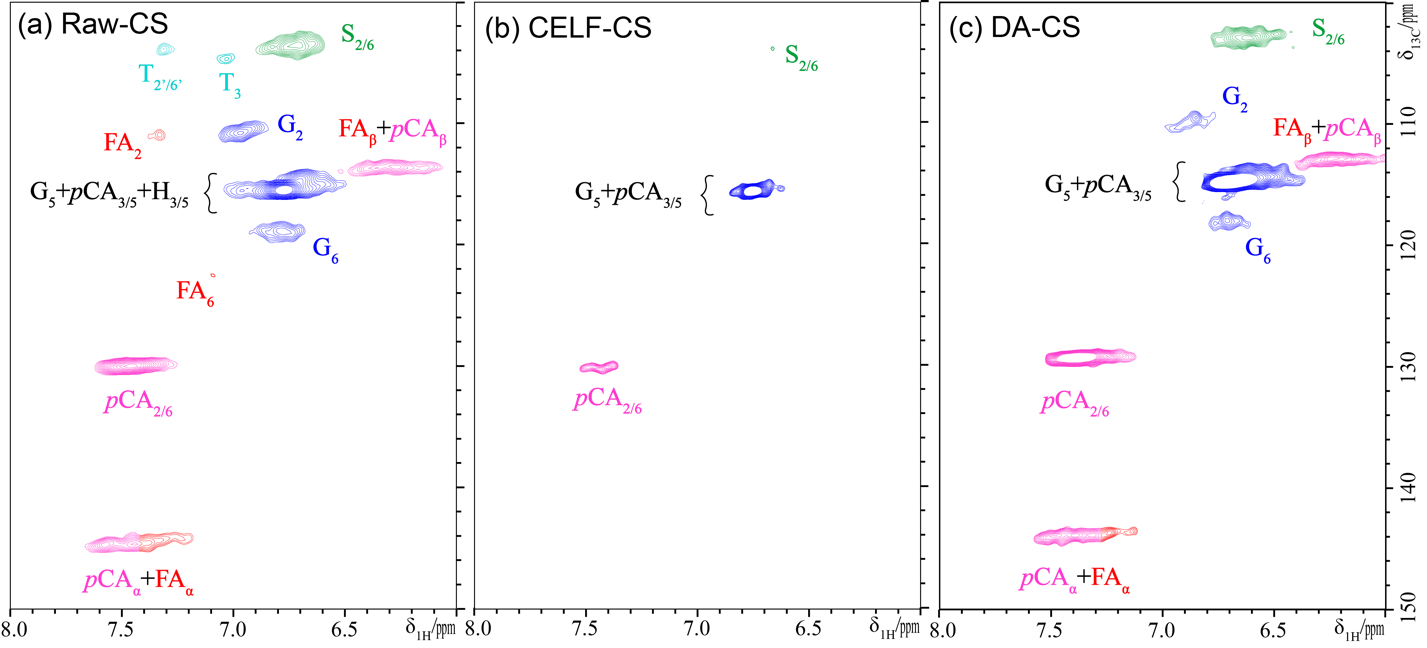

**Figure S6.** 2D NMR spectra of corn stover lignin revealing lignin subunits. Partial short-range ^13^C-^1^H (HSQC) correlation spectra (aromatic regions) of isolated lignin in DMSO-*_d_*_6_ from (a) raw corn stover (Raw-CS), (b) CELF pretreated corn stover (CELF-CS), and (c) DA pretreated corn stover (DA-CS). The structures are colored to match the contours assigned in the NMR spectra.


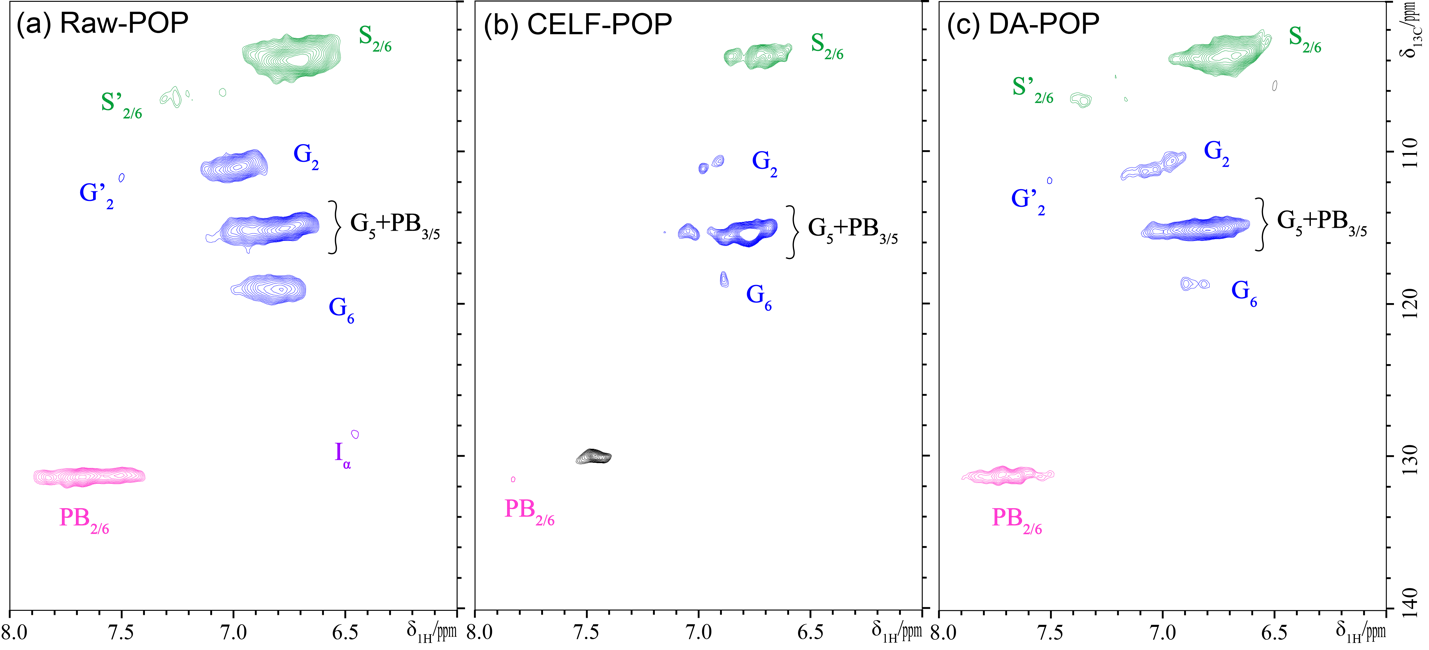

**Figure S7.** 2D NMR spectra of *Populus* lignin revealing lignin subunits. Partial short-range ^13^C-^1^H (HSQC) correlation spectra (aromatic regions) of isolated lignin in DMSO-*_d_*_6_ from (a) raw poplar (Raw-POP), (b) CELF pretreated poplar (CELF-POP), and (c) DA pretreated poplar (DA-POP). The structures are colored to match the contours assigned in the NMR spectra.

**References**

[S1] M. J. Selig, N. Weiss, and Y. Ji, “Enzymatic saccharification of lignocellulosic biomass,” NREL Laboratory Analytical Procedure, National Renewable Energy Laboratory, Golden, Colorado (2008).

[S2] D. Hogsett, “Cellulose hydrolysis and fermentation by *Clostridium thermocellum* for the production of ethanol,” PhD Thesis, Dartmouth College, (1995).

[S3] A. Sluiter, B. Hames, R. O. Ruiz, C. Scarlata, J. Sluiter, and D. Templeton, “Determination of structural carbohydrates and lignin in biomass,” NREL Laboratory Analytical Procedure, National Renewable Energy Laboratory, Golden, Colorado (2012).

[S4] M. Li, M. Tu, D. Cao, P. Bass, and S. Adhikari, “Distinct roles of residual xylan and lignin in limiting enzymatic hydrolysis of organosolv pretreated loblolly pine and sweetgum,” *J. Agric. Food Chem.* **61**(3), 646–654 (2013).

[S5] L. R. Lynd, P. J. Weimer, W. H. Van Zyl, S. Isak, and I. S. Pretorius, “Microbial cellulose utilization: Fundamentals and biotechnology of microbial cellulose utilization,” *Microbiol. Mol. Biol. Rev*. **66**(3), 506-577 (2002).

[S6] Ciesielski, P.N., Wang, W., Chen, X., Vinzant, T.B., Tucker, M.P., Decker, S.R., Himmel, M.E., Johnson, D.K., and Donohoe, B.S. “Effect of mechanical disruption on the effectiveness of three reactors used for dilute acid pretreatment of corn stover, Part 2: Morphological and structural substrate analysis,” *Biotechnology for Biofuels* **7**, 1–12 (2014).

[S7] B. S. Donohoe, P. N. Ciesielski, and T. B. Vinzant, “Preservation and preparation of lignocellulosic biomass samples for multi-scale microscopy analysis,” in *Biomass Conversion*, Humana Press, Totowa, NJ, **908**, 31–47 (2012).

[S8] C. G. Yoo, Y. Pu, M. Li, A. J. Ragauskas. “Elucidating structural characteristics of biomass using solution‐state 2 D NMR with a mixture of deuterated dimethylsulfoxide and hexamethylphosphoramide,” *ChemSusChem* **9**(10), 1090-1095 (2016).
